# Diversity and abundance of antimicrobial resistance genes in manure from pig farms with varying antibiotic use: a long-read metagenomic sequencing approach

**DOI:** 10.1186/s40813-026-00496-3

**Published:** 2026-02-27

**Authors:** Michael Biggel, Thomas Oberhänsli, Dolf Kümmerlen, Michael Walkenhorst, Roger Stephan, Mirjam Holinger

**Affiliations:** 1 Institute for Food Safety and Hygiene, Vetsuisse Faculty, University of Zurich, Winterthurerstrasse 272, Zurich, 8057 Switzerland; 2 FiBL Research Institute of Organic Agriculture, Ackerstrasse 113, Frick, 5070 Switzerland; 3 Division of Swine Medicine, Department for Farm Animals, Vetsuisse Faculty, University of Zurich, Winterthurerstrasse 260, Zurich, 8057 Switzerland

**Keywords:** Antimicrobial resistance (AMR), Long-read shotgun metagenomics, Pig manure, Mobile genetic elements (MGEs), Tigecycline resistance, Tet(X6)

## Abstract

**Background:**

Livestock production contributes to the emergence and spread of antimicrobial resistance (AMR), with pig farming accounting for a large share of veterinary antibiotic use. Manure application to fields can release drug-resistant bacteria and AMR genes into the environment, creating potential transmission routes to humans. Mobile genetic elements such as plasmids and transposons facilitate horizontal transfer of AMR genes between bacteria, including pathogens. However, quantitative data on the manure resistome and its links to antibiotic use remain limited. Shotgun metagenomics provides broad insights into microbiota and AMR composition, with long-read sequencing offering improved resolution of the genomic context of AMR genes. Here, we applied long-read shotgun metagenomics to investigate the diversity, abundance, and mobility potential of AMR genes in 24 manure samples from 14 Swiss pig farms with documented antibiotic use.

**Results:**

Across 24 manure samples, 225 distinct AMR genes were detected, with tetracycline resistance genes being most prevalent. Manure samples from farms reporting the highest recent antibiotic use contained greater AMR gene abundance and richness. Metagenomic assemblies revealed that 77% of AMR genes with resolved flanking regions were located near transposases, recombinases, integrases, or relaxases, suggesting high transfer potential. The tigecycline resistance gene *tet*(X6) and related variants were identified in 21 of 24 samples, frequently embedded within mobile genetic elements. Two samples contained complete gene clusters of the vancomycin resistance determinant *vanB*, one of which was part of the conjugative transposon *Tn*1549. In one sample, a single highly abundant plasmid encoding beta-lactam and aminoglycoside resistance accounted for 42% of the total AMR gene load.

**Conclusions:**

Pig manure is a reservoir of diverse and mobile AMR genes, including those conferring resistance to critically important antibiotics. Long-read metagenomics adds valuable genomic context, supporting AMR monitoring and risk assessment within a One Health framework.

**Supplementary Information:**

The online version contains supplementary material available at 10.1186/s40813-026-00496-3.

## Introduction

Antimicrobial resistance (AMR) is a major threat to global public health. While antibiotic use in humans is recognized as a key driver, animal production plays a significant role as well. The industrialization of livestock production has led to a surge in antibiotic consumption in animals, accounting for an estimated 70% of all antimicrobials sold by mass, predominantly for pig and cattle farming [1]. A major concern is the agricultural use of manure as fertilizer, which results in the massive release of drug-resistant bacteria and AMR genes into soils, water, and crops, with potential introduction into the human food chain. Many resistance genes are located on mobile genetic elements, which could facilitate their transfer to human pathogens [2].

Quantifying the impact of veterinary antibiotic use on drug-resistant infections in humans is challenging. Notable cases demonstrating strong links include the rise in ciprofloxacin-resistant *Campylobacter* and *Salmonella* infections in humans in the 1990s, which coincided with an increased use of fluoroquinolones in veterinary medicine [3, 4]. More recently, the emergence and spread of plasmid-mediated colistin resistance was strongly associated with colistin use in pigs [5]. A ban of colistin as animal feed additive in China in 2017 resulted in a drastically reduced prevalence of mobile colistin resistance in *Escherichia coli* in both animals and humans [6]. Similarly, the ban of the glycopeptide avoparcin as a growth promoter led to a rapid decline of vancomycin-resistant enterococci in farm animals [7].

Legislation on organic farming restricts the antibiotics use in animal husbandry to reduce the risk of AMR development, promote preventive measures, and encourage complementary medicine as first line treatment. The Swiss legislation permits a maximum of one antibiotic treatment for organic livestock with a lifespan under one year – such as fattening pigs – and up to three treatments per year for animals living longer than one year, such as sows [8]. Additionally, withdrawal periods must be twice as long as those on conventional farms. Analyses of treatment data have shown that these restrictions lead to lower antimicrobial use on organic pig farms [9] and a reduced abundance of resistant bacteria or AMR (reviewed by [10, 11]).

AMR monitoring is essential to detect emerging resistances and guide public health interventions. Key targets of surveillance efforts are AMR genes that confer resistance to critically important antibiotics used to treat multidrug-resistant infections. These include carbapenemases such as *bla*NDM or *bla*OXA-48, extended spectrum beta lactamases (ESBLs) such as *bla*CTX-M, plasmid-mediated tigecycline resistance mediated by Type B and Type C *tet*(X) variants, vancomycin resistance genes (*van*), or mobile colistin resistance (*mcr*) genes. Monitoring programs often rely on resistance data from culturable indicator bacteria like *E. coli* [12]. Quantitative PCR (qPCR) can be used as a culture-independent approach to study AMR gene abundances irrespective of the host but relies on and is biased by pre-defined targets [13]. Untargeted (“shotgun”) metagenomics involves the sequencing of the entire genetic material present in a sample. While short-read shotgun metagenomic sequencing has been widely used to determine the abundance and diversity of AMR genes, it usually falls short in resolving their genetic context. Recent advances in long-read sequencing technologies provide new opportunities, enabling detailed insights into the microbial community and mobile genetic elements carrying AMR genes [14].

In this study, we used long-read shotgun metagenomic sequencing to characterize the diversity and abundance of AMR genes in manure samples from 14 pig farms in Switzerland, with a focus on resistance to critically important antibiotics. We further investigated the relationship between antibiotic use and the abundance and diversity of AMR genes. These insights are important to evaluate interventions to limit AMR spread, and to advance metagenomic surveillance as a tool for AMR monitoring.

## Materials and methods

### Selection of farms and sample collection

Samples were collected between February and June 2023 on 14 pig farms across Switzerland (Table 1). Farms were selected to capture a range of production systems, including organic (*n* = 8) and conventional farms (*n* = 6), as well as fattening (*n* = 4), breeding (*n* = 5), and mixed-production types (*n* = 4) as well as one farm with pregnant sows only (*n* = 1). Within each production system, farms with low and high antimicrobial use in year 2022 were included to reflect different management practices. Actual antibiotic usage data within six weeks and six months before sample collection was used for correlation analyses. Organic farms produced according to the Swiss organic farming ordinance [8] and the private Bio Suisse guidelines [15].

**Table 1:** Characteristics of the 14 selected pig farms

Farm ID	Production system	Farm type	Number of pigs during sample collection	Sampling locations
A	Conventional	Mixed	50 sows; 280 fattening pigs	Drainage + storage
B	Organic	Breeding	20 sows	Drainage + storage
C	Conventional	Mixed	70 sows; 300 fattening pigs	Drainage + storage
D	Conventional	Breeding	130 sows	Drainage + storage
E	Organic	Fattening	200 fattening pigs	Drainage + storage
F	Organic	Mixed	130 sows; 200 fattening pigs	Drainage + storage
G	Organic	Mixed	50 sows; 300 fattening pigs	Drainage + storage
H	Conventional	Breeding	140 sows	Drainage + storage
I	Conventional	Fattening	180 fattening pigs	Drainage + storage
J	Organic	Breeding	50 sows	Drainage + storage
K	Organic	Fattening	180 fattening pigs	Drainage
L	Conventional	Fattening	100 fattening pigs	Drainage
M	Organic	Breeding	45 sows	Drainage
N	Organic	Pregnant sows	145 pregnant sows	Storage

Farmers were asked to stir the manure in the storage facility for 1–2 h before sample collection. We also ensured that the manure drainage system had not been flushed in the days before sample collection. Manure storage contained no manure from other livestock. At each location (manure drainage and manure storage), we collected three samples. If the drainage system was completely empty, pooled fecal samples were taken instead. Samples from the drainage system were collected using a scoop, while a bucket on a rope was used for samples from the manure storage. All sampling equipment was thoroughly cleaned with soap and lye between collections. On three farms (L, K, M), manure storage was not accessible; on one farm (N), no drainage sample was available. Manure samples were collected in 100 ml screw cap beakers (PS) and transported on ice to the laboratory. On the same day, equal portions of the three subsamples were pooled, aliquoted into 0.3 ml volumes in 1.5 ml tubes (PP), and stored at − 20 °C.

### Collection of antibiotic use data

Data on the use of antibiotics was extracted from electronic treatment journals (ETJ). The ETJ is used by pig farmers throughout Switzerland and recognized by veterinary authorities for mandatory documentation of treatments. For each antibiotic treatment, the ETJ records the preparation quantity, treatment date and duration, animal weight, indication, and pig category (suckling / weaned piglets, fattening pigs, pregnant / lactating sows). Active substances in the preparations and their concentrations are also recorded. For each herd, all antibiotic treatments within six weeks and six months prior to sampling were extracted. Antibiotic use was quantified as Defined Course Doses (DCD_ch_) per animal, where one DCD_ch_ represents the amount of active substance required for a complete treatment in a pig, based on Specific Product Characteristics (SPCs) of the antibiotic preparations authorised in Switzerland [16]. The number of DCD_ch_ per animal was calculated by dividing the total amount of active substance (mg) used in a pig category by the defined doses (DCD_ch_; mg/kg) multiplied by the standard weights (kg) of the different age groups as defined by the European Surveillance of Veterinary Antimicrobial Consumption (ESVAC) (piglets: 4 kg; weaners: 12 kg; finisher pig: 50 kg and sow: 220 kg).

### Shotgun metagenomic sequencing and data analyses

Manure samples (0.3 mL) were combined with 0.3 mL 2x DNA/RNA Shield (Zymo Research) and DNA extracted using the Quick-DNA HMW MagBead Kit (Zymo Research) including enzymatic lysis steps with lysozyme (final 10 mg/ml; Sigma-Aldrich) and MetaPolyzyme (0.25 mg/ml; Sigma-Aldrich). Libraries were prepared using the Ligation Sequencing Kit V14 (SQK-LSK114, Oxford Nanopore Technologies) and sequenced on R10.4.1 MinION flow cells (Oxford Nanopore Technologies) with one sample per flow cell. Basecalling was performed using guppy 6.1.1 (https://nanoporetech.com/document/Guppy-protocol) in combination with model dna_r10.4.1_e8.2_400bps_sup@v4.2.0 and a minimum quality score of 10. Reads were filtered to a minimum length of 200 bp using nanoq 0.10.0 [17]. Reads mapping to the Sus scrofa host genome GCF_000003025.6 were discarded using minimap2 v2.17 [18] (option -ax map-ont) and samtools v1.7 [19]. SingleM v0.18.3 [20] (options --hmmsearch-package-assignment --min-taxon-coverage 0.35) in combination with v4.3.0 (GTDB 09-RS220) metapackage was used for taxonomic profiling and to estimate the total number of cells represented in each dataset (full coverage root). AMR gene screening was conducted using a custom database (NCBI + *tet*) comprising the NCBI AMRFinderPlus core database (version 2024-12-18.1) [21] supplemented additional with *tet*(X) variants X6 to X57 obtained from the database described by Yao et al. [22]. To estimate AMR gene abundances, sequencing reads were mapped to the NCBI + *tet* database using minimap2 v2.17 [18] with the parameters -ax map-ont --sam-hit-only. SAM files were converted to BAM format using samtools v1.7 [19], excluding unmapped reads and secondary alignments. Coverage statistics for each AMR gene was calculated using pileup.sh from the BBMap suite v39.2 [23]. Hits with < 80% template coverage or an average read depth < 2 were excluded. Final coverage values were normalized by estimated total cell numbers determined with SingleM.

To assess whether sequencing depth was sufficient to capture AMR gene diversity, rarefaction analyses were performed for each sample. Reads were subsampled to increasing sequencing depths using rasusa 2.1.0 [24], and subsampled reads were mapped to the AMR gene database and filtered (template coverage ≥ 80%, average read depth ≥ 2) as described above. The cumulative number of unique AMR genes detected at each depth was recorded. Rarefaction curves were fitted using a saturating non-linear model of the form y = (A * x)/ (B + x), where y is the number of detected AMR genes at sequencing depth x (Gbp), A is the estimated asymptotic AMR gene richness, and B is the half-saturation constant, i.e., the sequencing depth at which half of the asymptotic diversity is recovered. Curve fitting was performed using non-linear least squares regression (scipy.optimize.curve_fit in python). Observed AMR gene richness, i.e., the total count of unique AMR genes detected in a sample after standardization, was reported for sequencing data rarefied to 7 Gbp, corresponding to the lowest sequencing depth across samples. Alpha diversity of AMR genes was calculated for each sample using the diversity() function of the vegan R package (v2.7).

Metagenome assemblies were generated using flye v2.9.5 [25] (options --nano-hq --meta --iterations 1). AMR genes in metagenomic assemblies were detected using abricate v1.0.1 (https://github.com/tseemann/abricate) (options --mincov 70 --minid 90) in combination with the NCBI + *tet* database. Selected contigs were extracted using SeqKit v2.10.0 [26] and annotated using Bakta 1.11.0 [27]. Contig lengths were determined using SeqKit v2.10.0 [26]. Taxonomic classification of contigs was attempted using Kraken2 v2.1.6 (--confidence 0.51) [28] with the Standard Kraken 2 database (downloaded in November 2025). A database of genes associated with AMR gene transfer was constructed by retrieving bacterial transposases, integrases, recombinases, or relaxases from UniProt’s UniRef90 database [29] (accessed 09/04/2025), excluding sequences shorter than 100 amino acids. The resulting database – comprising 826,446 transposases, 383,396 integrases, 364,683 recombinases, and 44,460 relaxases – was used to screen metagenomic assemblies with DIAMOND v2.1.11 [30] (parameters --query-gencode 11 --id 60 --subject-cover 60 --frameshift 15 --max-target-seqs 1 --range-culling --sensitive --max-hsps 0). AMR genes were considered likely transferable if their start or end position was within 5,000 bp of the start or end of a transposase, integrase, recombinase, or relaxase gene. AMR genes with unresolved flanking regions, i.e., located near (< 5,000 bp) contig ends, were excluded from the analysis. Heavy metal resistance genes were detected in metagenomic assemblies using the BacMet 2.0 database (155,512 predicted resistance genes) in combination with DIAMOND v2.1.11 [30] (parameters --query-gencode 11 --id 70 --subject-cover 90 --frameshift 15 --max-target-seqs 1 --range-culling --sensitive --max-hsps 0).

Clustal Omega [31] was used for gene and protein sequence alignments. Genetic structures were compared and visualized using clinker v0.0.31 [32]. Associations between antibiotic use and AMR gene abundance were assessed with Kendall’s rank correlation coefficient (τ), using the cor.test function in R [33]. To compare AMR gene abundance between groups, a Wilcoxon rank-sum test was performed using the wilcox.test function. A two-sided *p*-value of less than 0.05 was considered statistically significant.

## Results

### Diversity, abundance, and mobility of AMR genes

We analyzed 24 manure samples from 14 pig farms with known differences in antibiotic use. Samples were collected from the manure storage tanks (*n* = 11) and drainage systems (*n* = 13) and subjected to shotgun metagenomic sequencing. After removal of pig-derived reads (0.1 to 0.4% of total) and quality filtering, 7.1 to 13.4 Gbp of data per sample were available for analysis (Supplementary Table S1). Rarefaction analyses indicated that our data captured between 38% and 87% of the estimated total AMR gene diversity (richness), with most samples reaching 65–80% of their predicted asymptote (Supplementary Fig. 1).

**Fig. 1:**
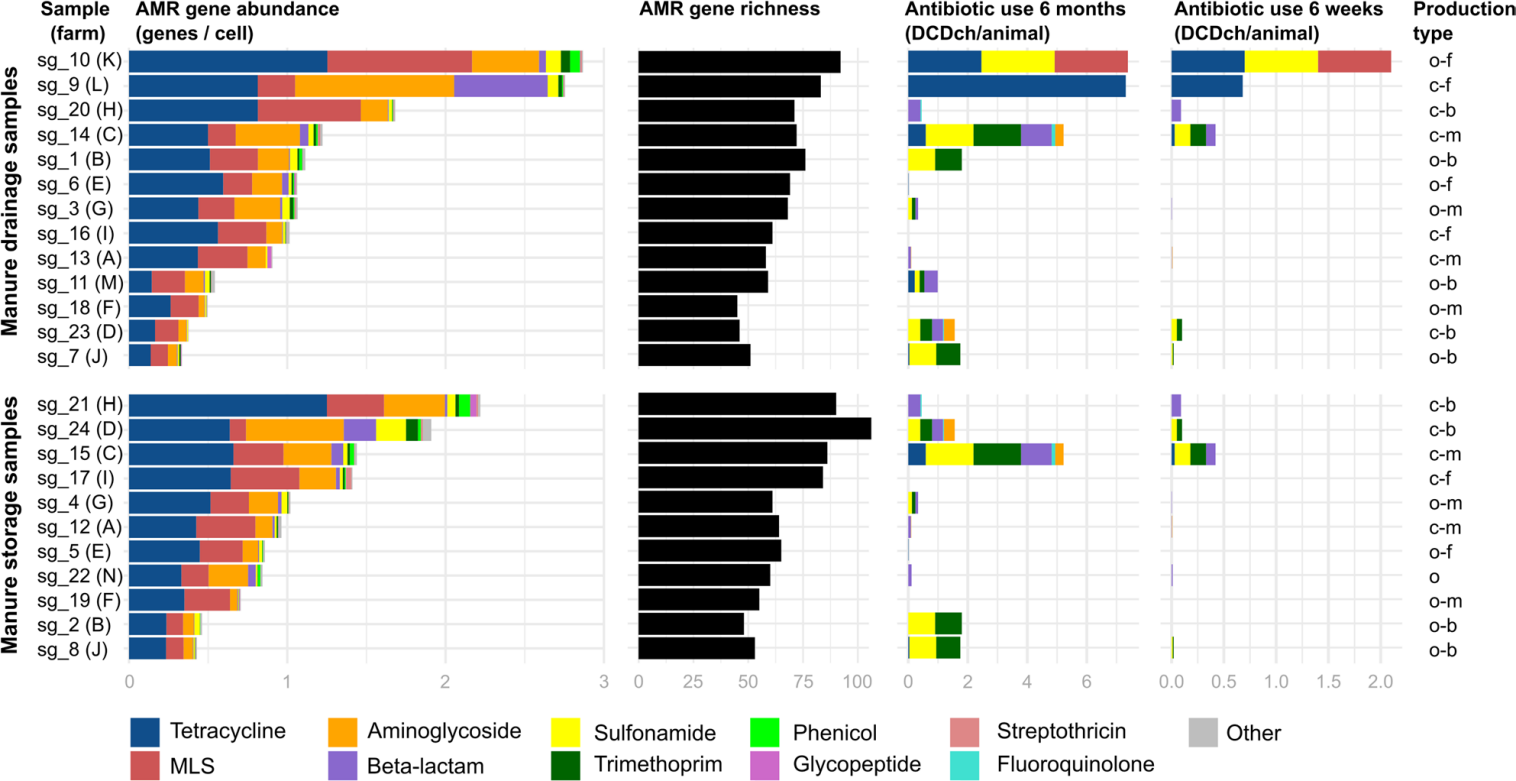
Relative abundance and observed richness of antimicrobial resistance genes in manure samples and corresponding antibiotic use across 14 farms. Richness corresponds to the number of distinct AMR genes detected in rarefied data. Antibiotic use is shown for the six weeks and six months preceding sample collection. Ten farms (A to J) were sampled at both the manure drainage system and storage tanks. Production types: c: conventional, o: organic, b: breeding, f: fattening, m: mixed type (fattening and breeding). DCDch/animal: Defined Course Doses per animal, based on the Swiss standard for veterinary antimicrobial treatments

**Fig. 2:**
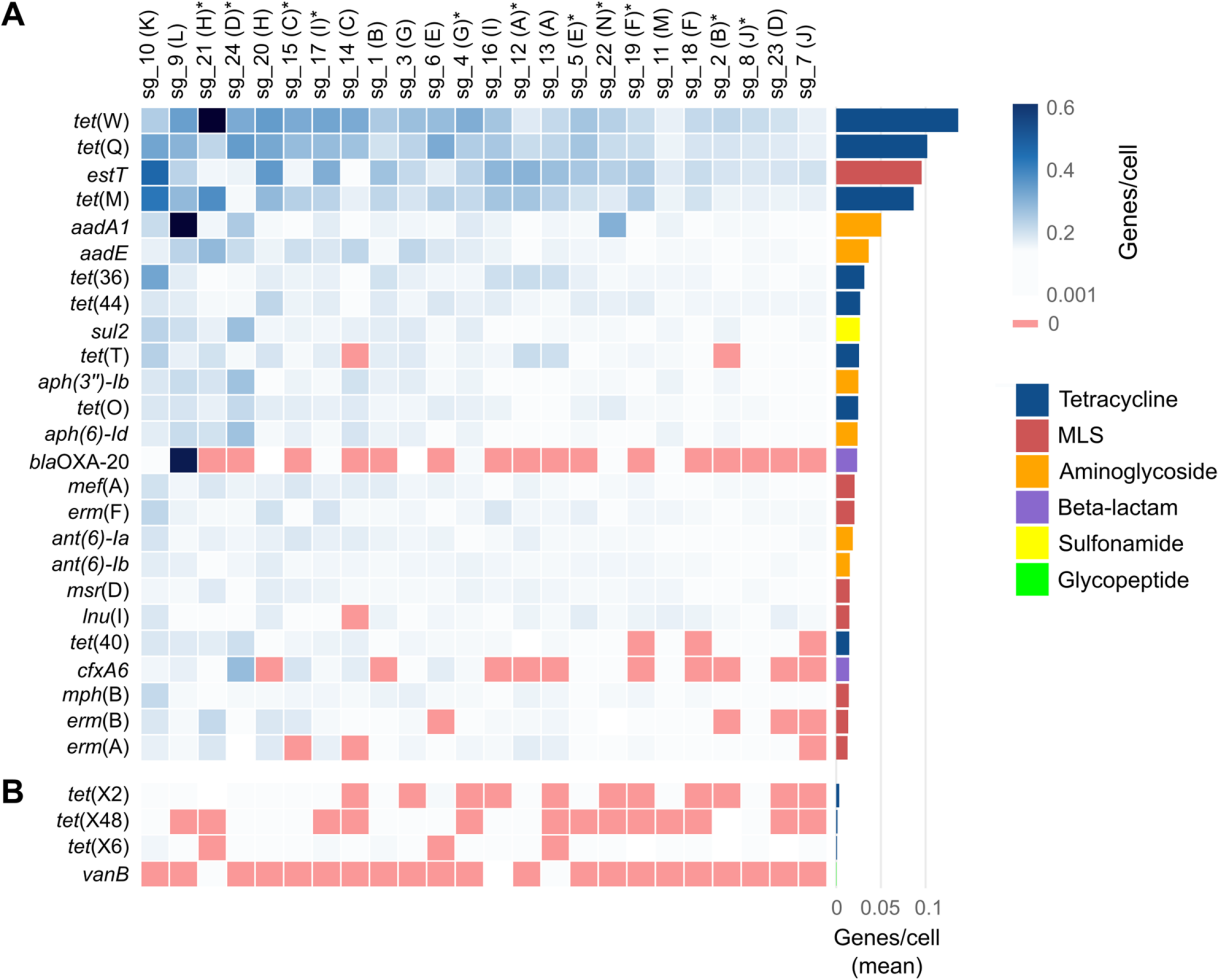
Normalized abundance of selected antimicrobial resistance genes across 24 samples. (**A**) Top 25 most abundant AMR genes. (**B**) Selected AMR genes discussed in the results section. Red fields in the heatmap indicate that a gene was not detected. The barplot shows the mean abundance of each gene across all samples. Farms are labelled A to N, with manure storage samples marked by an asterisk. MLS: macrolide-lincosamide-streptogramin group

A read mapping-based approach identified 225 distinct AMR genes across the 24 samples (Supplementary Table S2). Tetracycline resistance genes were the most abundant, averaging 0.5 genes per cell (i.e., per genome equivalent) and accounting for 44.4% of the total AMR gene load (Fig. 1), with *tet*(W) (0.14 genes/cell) and *tet*(Q) (0.10 genes/cell) being the most abundant AMR genes overall (Fig. 2). Notably, the type C *tet*(X) variant *tet*(X6) encoding high-level tigecycline resistance was detected in 21 of 24 samples, reaching up to 0.019 genes/cell in sample sg_10. Additional detected *tet*(X) variants included *tet*(X2) (type A, tigecycline inactive; 13/24 samples) and *tet*(X48) (type B, intermediate tigecycline resistance; 11/24 samples).

Genes associated with resistance to macrolide-lincosamide-streptogramin (MLS) antibiotics (0.28 genes/cell) and aminoglycosides (0.23 genes/cell) were also prominent. MLS resistance was primarily due to a high load of the recently described macrolide esterase gene *estT* (0.10 genes/cell), the third most common AMR gene overall. Beta-lactam and sulfonamide AMR genes were less abundant (0.05 and 0.03 genes/cell, respectively), while genes associated with resistance to trimethoprim, phenicol, streptothricin, and glycopeptide (*van* genes) were detected only sporadically (0.005 to 0.013 genes/cell). No genes linked to colistin or quinolone resistance were found.

In metagenomic assemblies, 148 distinct AMR genes were detected across all samples when applying strict BLAST thresholds (≥ 70% coverage, ≥ 90 identity). Several genes appeared in multiple distinct genetic contexts within the same assembly – up to 29 for *estT* in sample sg_10 (Supplementary Table S4). Tigecycline resistance genes *tet*(X6), *tet*(X6)-like (96.9 to 99.9% identity to *tet*(X6)), or *tet*(X48)-like (94.4 to 99.3% identity to *tet*(X48)) were detected in 13 of 24 assemblies in a total of 19 distinct genetic contexts. Of those, 13 encoded variants with five amino acid substitutions associated with tigecycline resistance (Met329, Thr339, Asn340, Ile350, and Glu351) (Cheng et al., 2022), while the remaining six carried three of the five substitutions (Supplementary Fig. 2).

**Fig. 3:**
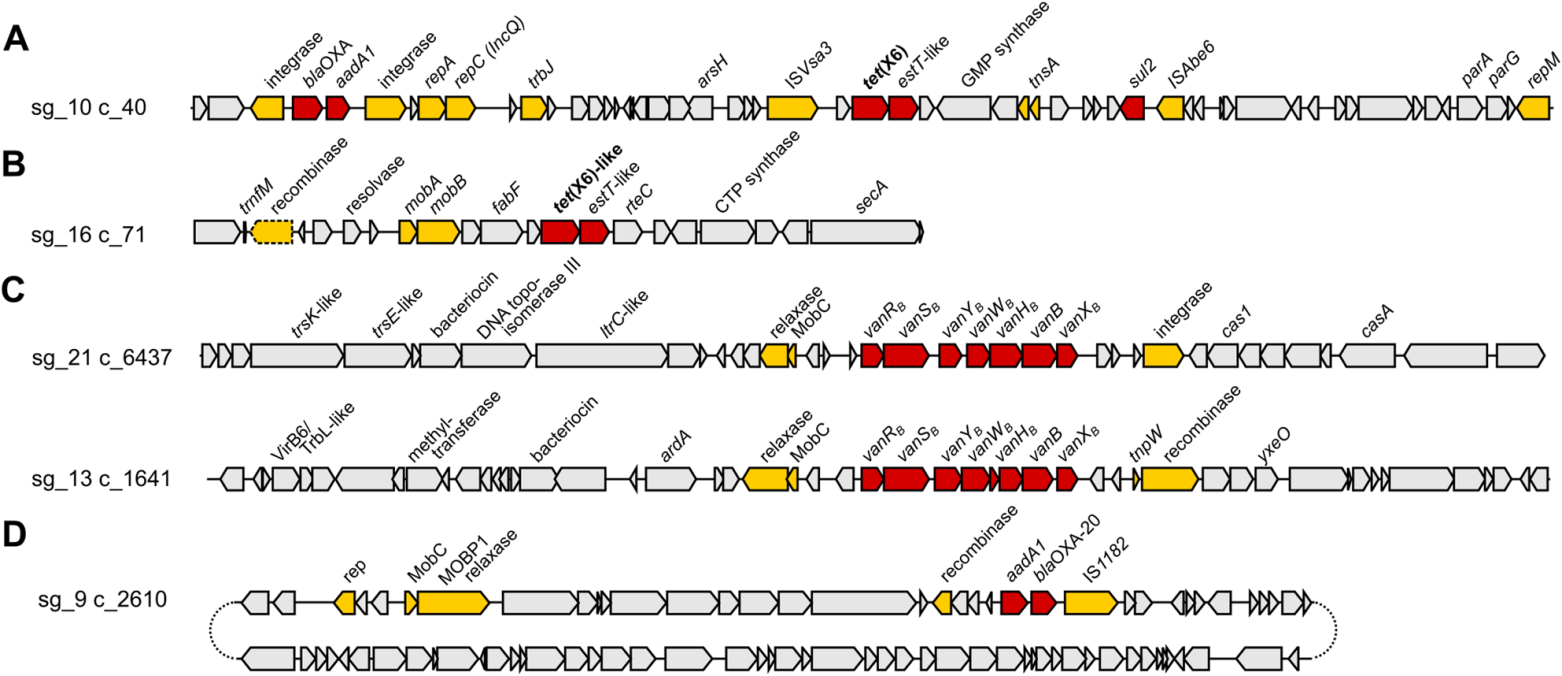
Genetic context of selected AMR genes in metagenomic assemblies from manure samples. (**A**) and (**B**) show the genetic surroundings of *tet*(X6) and a *tet*(X6)-like gene (99.9% identity) in sg_10 and sg_16, located on a putative conjugative plasmid and a putative integrative mobilizable element, respectively. (**C**) Genetic context of the *vanB* gene cluster. In assembly sg_21, *vanB* was part of transposon Tn*1549/*Tn*5382*. (**D**) Structure of a highly abundant resistance plasmid identified in sample sg_9. The plasmid was circularized through assembly. AMR genes are shown in red; genes related to transposition, integration, and plasmid replication or mobilization are shown in yellow

The *tet*(X6), *tet*(X6)-like, and *tet*(X48)-like genes were consistently located upstream of a macrolide esterase gene (79 to 84% identity to EstT [UXD71803.1]) and frequently near transposase (IS*Vsa3*) or relaxase (MobB) genes, suggesting transferability (Supplementary Fig. 3). One *tet*(X6) carrying contig harbored additional AMR genes (*bla*OXA, *aadA1*, *sul2*) and multiple plasmid-associated genes (*repA*, *repC*, *trb*, *parA*, *repM*) matching those frequently found in Gammaproteobacteria (Fig. 3A). Most *tet*(X6)-like genes were embedded within the conserved genetic structure *recombinase*-*mobA*-*mobB*-*fabF*-*tet*(X6)-like-*estT*-like-*rteC* (Fig. 3B, Supplementary Fig. 3). This putative integrative mobilizable element (IME) was typically integrated into the chromosome at tRNA sites. Although the genetic surrounding of the *tet*(X6)-like genes was often well assembled (median contig length 21 kb, range 3 kb to 1.04 Mbp), most contigs could not be taxonomically classified with Kraken2. Where classification was possible, assignments were typically limited to the kingdom level (Pseudomonadati), with only occasional resolution to the phylum level (Bacteroidota) (Supplementary Fig. 3), suggesting that the contigs originate from species not represented in the database.

No extended-spectrum beta lactamase (ESBL) or carbapenemase genes were identified in any assembly, except for *bla*ACI-1, found in four samples. Two metagenomic assemblies (sg_13 and sg_21) harbored complete *vanB* gene clusters. In sg_21, this cluster was located on the conjugative transposon Tn*1549/*Tn*5382* (Fig. 3C), a frequent carrier of VanB-type vancomycin resistance in Enterococci. In sg_13, *vanB* was found in an uncharacterized genomic context near a TrbL/VirB6 plasmid conjugal transfer protein. Genomic regions distant to *vanB* showed no homology to Enterococci or other genomes in NCBI’s core nucleotide database.

Across all samples, 829 of the 2008 AMR gene hits had resolved flanking regions. Of these, 638 (77.0%) were located within 5,000 bp of genes encoding transposases, recombinases, integrases, or relaxases, suggesting horizontal acquisition (Supplementary Table S5). Among the 98 unique AMR genes with resolved context, 84 (89.4%) were located near mobility-associated genes in at least one contig. The most frequently detected genes involved in mobilization were an uncharacterized recombinase (Uniprot A0A848BA12; *n* = 25) and a relaxase (A0A1M5IKG7; *n* = 20), which were often found near *tet*(W) and *tet*(36), respectively. In addition, 273 (32.9%) of the 829 genes were near (< 5,000 bp) other AMR genes, suggesting frequent co-transfer of AMR genes. The most frequently co-located gene pairs were *mef(A)* + *msr(D)* (macrolide resistance; *n* = 18) and *aph(3’’)-Ib* + *aph*(6)*-Id* (aminoglycoside resistance; *n* = 17) (Supplementary Table S5). Known heavy metal resistance genes were found near AMR genes in only 7 of 829 cases (0.8%), specifically adjacent to *aadA1*,* aph*(6)*-Id*, *aph(3’’)-Ib*, or *sul1* (Supplementary Table S6).

### Contribution of individual mobile genetic elements to AMR gene abundances

In the manure drainage sample sg_9 from farm L, the abundance of aminoglycoside and beta-lactam resistance genes was 5.1-fold and 22.3-fold higher, respectively, than the mean across all other samples (Fig. 1). The metagenomic assembly of this sample revealed a highly abundant (0.53 copies/cell) 63.7 kb plasmid carrying a *bla*OXA-20-like gene (97% amino acid sequence identity; beta-lactam resistance) and an *aadA1*-like gene (95% amino acid sequence identity; aminoglycoside resistance) (Fig. 3D). The plasmid accounted for 41.8% of the total AMR gene load in sg_9: 96.9% of the beta-lactam and 57.7% of the aminoglycoside AMR gene abundance. BLAST searches against NCBI‘s nucleotide database revealed high similarity (> 90% sequence coverage and identity) between the plasmid’s relaxase and mobilization protein MobC and homologs from *Marinospirillum* sp., suggesting this genus as a potential host. Notably, *Marinospirillum* – detected in 19 of the 24 samples (Supplementary Table S7) – was among the dominant genera in sample sg_9, with a relative abundance of 4.8% (Supplementary Fig. 4). No similar plasmids were identified in NCBI‘s nucleotide database or in other samples. However, the putative transposition unit recombinase - *aadA1*-like - *bla*OXA-20-like - IS*1182* was also detected in the metagenomic assembly of sample sg_10, but in a distinct genetic context.

A similarly high abundance of a single resistance gene was only observed for *tet*(W) in sample sg_21, reaching 0.61 genes/cell (Fig. 2). Unlike *bla*OXA-20 and *aadA1* in sg_9, *tet*(W) was found in 12 distinct genetic contexts (contigs) in the metagenomic assembly of sg_21, suggesting its presence across multiple strains.

### Correlations between antibiotic use, AMR gene abundance, and AMR gene richness

Antibiotic use during the six weeks before sampling ranged from 0.0 to 2.1 DCD_ch_/animal (mean: 0.244 DCD_ch_/animal), with five farms reporting no antibiotic use. The most frequently administered antibiotics were tetracyclines (0.101 DCD_ch_/animal), sulfonamides (0.064), macrolides (0.050), trimethoprim (0.014), and penicillin (0.014) (Fig. 1), while aminoglycosides (0.0003) and fluoroquinolones (0.0001) were rarely used. Notably, macrolides were used exclusively at farm K. Over the six months before sampling, antibiotic use ranged from 0.0 to 7.4 DCD_ch_/animal (mean: 1.93 DCD_ch_/animal), with two farms (I and F) reporting no antibiotic use.

Correlations between antibiotic use and AMR gene abundance and richness were analyzed separately for manure drainage (*n* = 13) and manure storage samples (*n* = 11). The two farms with the highest antibiotic use in the six weeks before sampling (K and L) exhibited the highest AMR gene abundances and richness in drainage samples (Fig. 1). Similarly, the three manure storage samples with the highest AMR gene abundance and richness originated from farms C, D, and H, which had the highest antibiotic use during that period.

Overall, Kendall’s rank correlation analysis indicated a moderate positive association between antibiotic use (six weeks) and AMR gene abundance among both storage samples (Kendall’s τ = 0.29) and drainage samples (Kendall’s τ = 0.36). While these associations were not statistically significant (*p* = 0.2 and *p* = 0.1, respectively), the trend suggests that larger sample sizes may be needed to detect robust effects. When antibiotic use was considered over a six months period, the correlation for drainage samples remained moderate (Kendall’s τ = 0.37, *p* = 0.08, whereas no association was observed for storage samples (Kendall’s τ = 0.07, *p* = 0.8).

Among drainage samples, the highest abundances of tetracycline, MLS, and sulfonamide resistance genes were detected in the sample from farm K, which had the highest usage (six weeks) of these three antibiotics (Fig. 1). Overall, we however found no strong correlation between specific antibiotic classes and the abundance of associated resistance genes. In drainage samples, tetracycline (τ = 0.45, *p* = 0.06) and MLS (τ = 0.39, *p* = 0.11) showed the strongest correlation. In storage samples, tetracycline (τ = 0.34, *p* = 0.21) and beta-lactam (τ = 0.26, *p* = 0.28) showed the strongest correlation.

Among paired samples from farms with similar production systems but substantial differences in antibiotic use in the six weeks prior to sampling (organic-fattening: sg_10 vs. sg_6; conventional-fattening: sg_9 vs. sg_16; conventional-mixed: sg_14 vs. sg_13, sg_15 vs. sg_12), AMR gene abundance was consistently higher on farms with greater antibiotic use. Our data showed a strong correlation between AMR gene abundance and AMR gene richness across both storage (τ = 0.79, *p* < 0.001) and drainage samples (τ = 0.86, *p* < 0.001). Consequently, correlation analyses between antibiotic use and AMR gene richness revealed similar patterns to those observed with AMR gene abundance, with moderate positive but statistically non-significant associations. The Simpson diversity index ranged from 0.89 to 0.97 across all samples, suggesting that the resistomes were generally even and diverse.

## Discussion

The widespread use of antibiotics in livestock production raises concerns about the release of AMR genes into the environment and the food chain. Consistent with previous studies, our analysis of pig manure revealed a high diversity and abundance of AMR genes – even on farms that had not used antibiotics during the six months before sampling. Our long-read sequencing approach offered improved insights into their genetic environment, often allowing AMR genes to be linked to mobilization markers or specific mobile genetic elements. A total of 89.4% of the identified unique AMR genes and 77% of all AMR gene occurrences (including repeated instances in different contigs or samples) were flanked by mobilization-associated genes, suggesting horizontal acquisition rather than intrinsic carriage by their host bacteria and a generally high potential for transferability of AMR genes in animal fecal samples. Given that our analysis relied on a database of known mobilization markers, the actual proportion of mobile AMR genes is likely even higher.

Notably, most samples contained genes linked to tigecycline inactivation – a last-resort tetracycline antibiotic used against multi-drug resistant pathogens. The highest abundance and diversity of tigecycline active *tet*(X) variants was observed in a sample from the farm reporting the highest tetracycline use during six weeks before sample collection. The presence of *tet*(X6) and closely related *tet*(X) variants were confirmed in metagenomic assemblies, where they were typically flanked by mobilization-associated genes. The *tet*(X6) gene was first reported in 2020 in a *Myroides phaeus* isolate from a pig fecal sample and has meanwhile been identified in various species including *Acinetobacter* spp., *Escherichia coli*, and *Proteus* spp [34, 35].

Two metagenomic assemblies contained complete *vanB* gene clusters, a major determinant of vancomycin resistance in enterococci species. In one case, the cluster was located on the widespread conjugative transposon Tn*1549/*Tn*5382*, which has the demonstrated ability to transfer from commensal anaerobes to clinically relevant *Enterococci* within in the murine gut [36]. The detection of *vanB* is unexpected, as the use of glycopeptides, including vancomycin and the growth promoter avoparcin, has been banned in Switzerland and the European Union due to concerns about vancomycin resistance [7]. Nevertheless, vancomycin-resistant enterococci continue to be detected in Swiss livestock and the environment [37, 38]. This persistence may be driven by co-selection pressures, as resistance genes often co-occur on mobile genetic elements with heavy metal resistance determinants, reflecting the continued use of copper and zinc as feed additives in livestock farming [37, 38]. In our metagenomic assemblies, no heavy metal resistance genes were detected near *vanB*, and such genes were only sporadically observed in the vicinity of other AMR genes. Genes encoding resistance to other critically important antibiotics, including extended-spectrum beta-lactamases (ESBLs), carbapenemases, or colistin resistance, were not detected. Genes present in low-abundance (< 0.1%) bacterial strains may however not be captured at the targeted sequencing depth.

In line with previous studies investigating AMR gene abundances in pig fecal and manure samples, genes associated with tetracycline, macrolide, and aminoglycoside resistance were overall predominant [39, 40]. Notably, the observed macrolide resistance was largely attributable to the recently described gene *estT* [41], highlighting how the choice of database and its version can substantially influence AMR gene quantification. Moreover, individual mobile genetic elements had a substantial impact on AMR abundance estimates. In one manure drainage sample, a single highly abundant plasmid encoding both aminoglycoside and beta-lactam resistance genes accounted for nearly half of the total observed AMR gene load. Although there was no reported use of aminoglycosides or beta-lactams on this farm, potential antibiotic-driven co-selection of this plasmid mediated by chromosomally encoded antimicrobial or heavy metal resistance genes cannot be ruled out.

Predicting AMR gene abundance based on antibiotic use remains challenging due to co-selection, mutation-driven resistance mechanisms, varying pharmacokinetics and selection pressures across antibiotic classes, and database biases toward clinically relevant resistance genes. While our limited sample size precluded robust correlation analyses, we observed substantially higher AMR gene loads in manure from the farms with the highest antibiotic use. This observation is consistent with previous studies: for instance, Munk et al. [39] reported a strong link between total national antibiotic use in livestock and AMR gene abundance in pooled pig fecal samples across 181 pig farms from nine European countries. Similarly, Andersen et al. [42] found a linear relationship between the lifetime use of most antibiotic classes (aminoglycoside, extended-spectrum penicillin, lincosamide, macrolide, and tetracycline) and AMR gene abundances in 83 fecal pig batch samples in Denmark. Interestingly, in our data AMR gene abundance was strongly correlated with AMR gene richness, suggesting that increased selection pressure may promote not only higher loads but also a broader diversity of resistance genes.

Notably, taxonomic profiling suggested the presence of *Marinospirillum* in most manure samples, including one where it was potentially the host of a highly abundant resistance plasmid. This genus is known to occur in saline or hypersaline environments including seawater, saline lakes, and fermented seafood [43–45] but has not been reported in manure or animal samples. It may have been introduced through environmental or feed-associated sources; alternatively, manure-associated environments may represent an under-recognized niche for this genus. Our attempts to isolate *Marinospirillum* using selective media however failed.

We acknowledge several limitations of our study. First, the low number of farms and samples precluded robust statistical analyses of correlations between AMR abundance and antibiotic use, and potential influences of production types could not be assessed. Second, it is common practice in many livestock farms for household wastewater to enter manure storage. Although the proportion of human-derived material is small relative to animal feces, we cannot fully exclude a potential contribution to the manure microbial community. Third, residual antibiotic concentrations in manure were not measured and should be assessed in future studies to better understand their effects. Finally, rarefaction analyses indicated that the sequencing depth achieved was insufficient to capture the full diversity of AMR genes.

In summary, our study shows the potential of long-read metagenomics to explore the diversity, abundance, and mobility of AMR genes. Gaining insight into the reservoirs and transferability potential of critical resistance genes is essential for guiding efforts to limit their spread.

## Supplementary Information

Below is the link to the electronic supplementary material.

Supplementary Material 1

Supplementary Material 2

## Data Availability

The datasets generated during this study are available in the NCBI Sequence Read Archive (SRA) under BioProject accession [PRJNA1279599] (https://www.ncbi.nlm.nih.gov/bioproject/PRJNA1279599) .
