# Supplementary material for: Diversity and abundance of antimicrobial resistance genes in manure from pig farms with varying antibiotic use: a long-read metagenomic sequencing approach
Source: Porcine Health Manag. 2026 Feb 27;12:15. doi: 10.1186/s40813-026-00496-3 (PMC13049923; doi:10.1186/s40813-026-00496-3)
Supplement: Supplementary file 2 — Supplementary Material 2 [file 40813_2026_496_MOESM2_ESM.docx]

**Supplementary Figures**

**
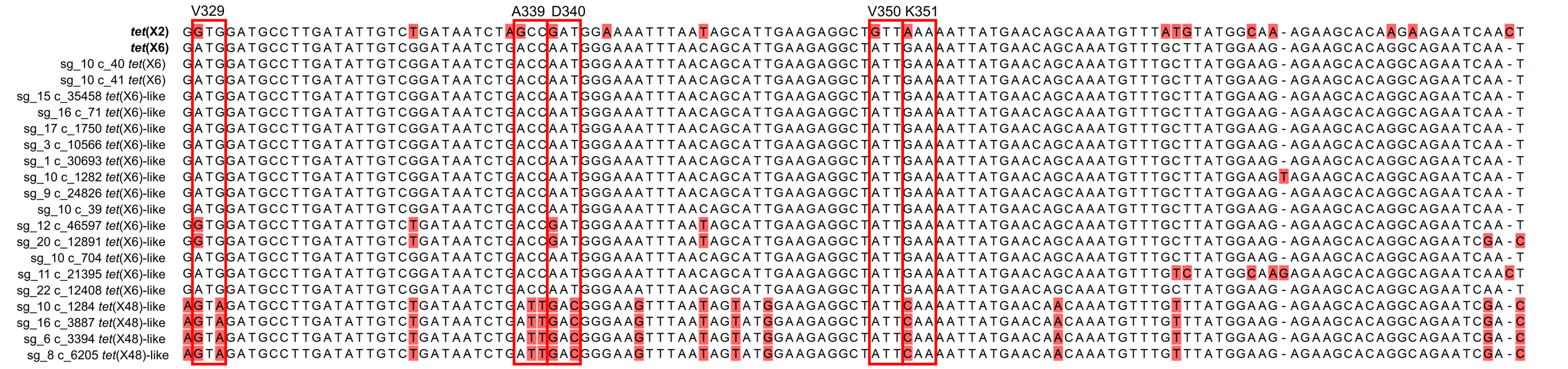
**

**
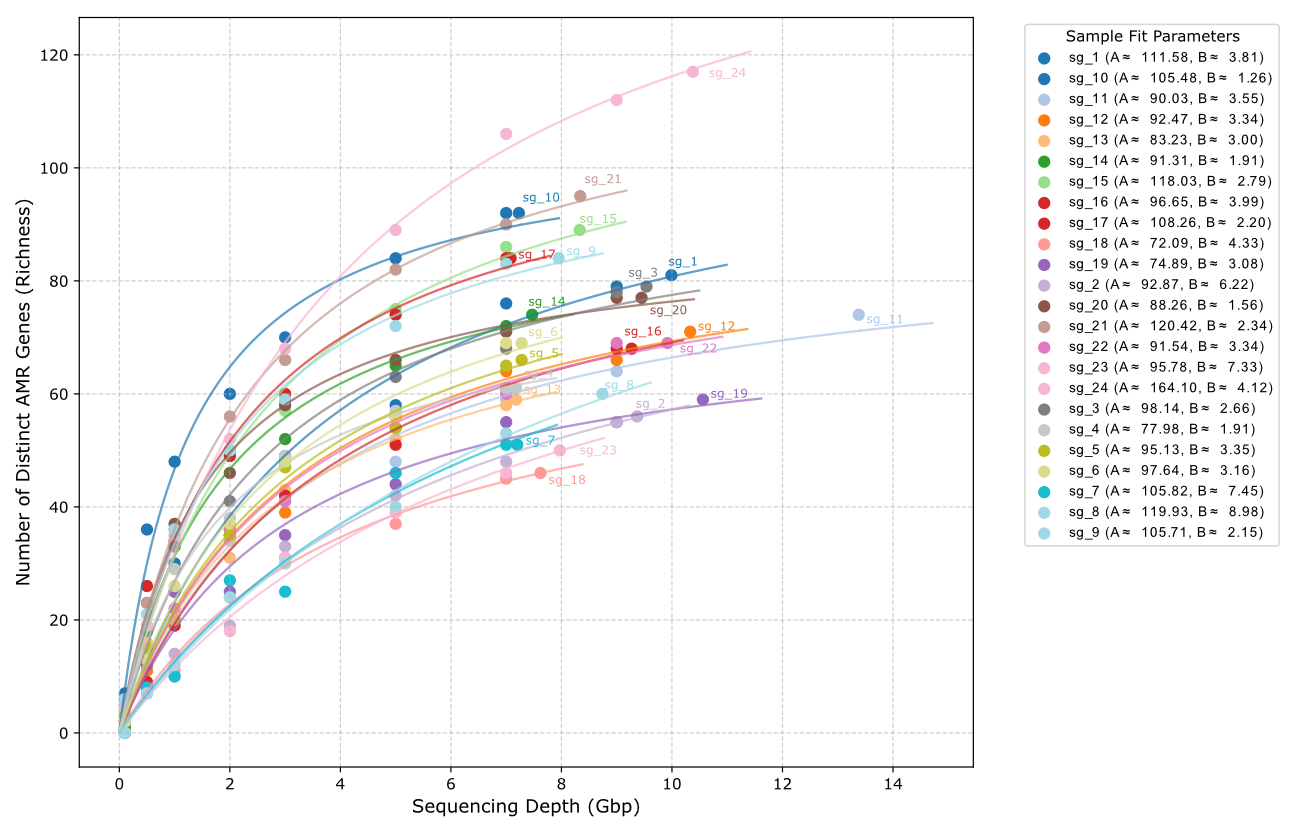
**

**Supplementary Figure 1**. Rarefaction analysis of AMR gene richness using the hyperbolic saturation model. Rarefaction curves were generated for all 24 metagenomic samples to model the relationship between sequencing depth and observed AMR gene richness. Observed data points are shown as colored circles, and the solid lines represent the fitted hyperbolic saturation curve. Estimated parameters based on the curve fitting are reported in the legend: A (asymptote): estimated maximum richness (total number of AMR genes) potentially present in the sample; B (half-saturation constant): sequencing depth (Gbp) required to detect approximately half of the estimated maximum richness.

**
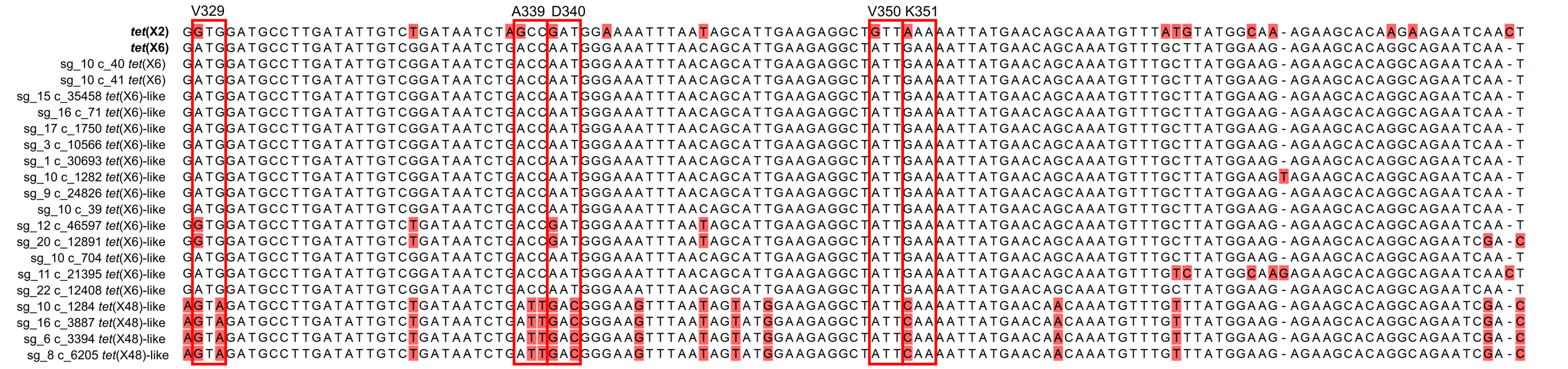
**

**Supplementary Figure 2**. Alignment of the putative tigecycline resistance-determining region from 19 *tet*(X) genes identified in metagenomic assemblies. The alignment includes reference sequences of the variants *tet*(X2) (inactive against tigecycline) and *tet*(X6) (tigecycline active). Amino acid positions previously associated with tigecycline activity (V329, A339, D340, V350, K351) are highlighted with a red box.

**
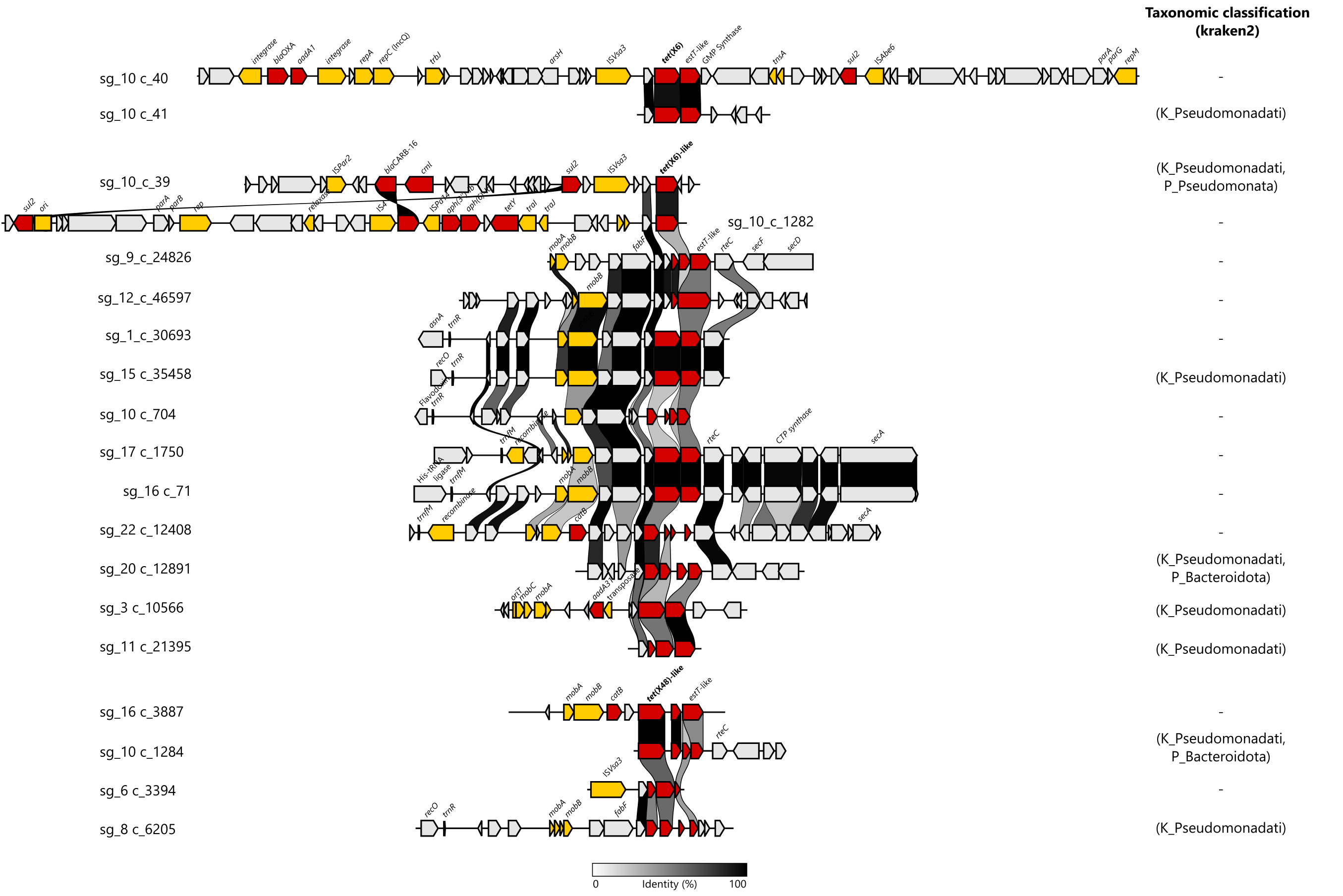
Supplementary Figure 3**. Genetic context of *tet*(X6), *tet*(X6)-like, and *tet*(X48)-like genes in metagenomic assemblies. AMR genes are labelled in red; genes involved in transposition, integration, plasmid replication, and mobilization in yellow. In low-coverage contigs, *tet*(X) and their neighboring genes were occasionally frameshifted, resulting in incomplete open reading frames. The kingdom and lowest resolved taxonomic rank are shown where kraken2 classification was possible.**
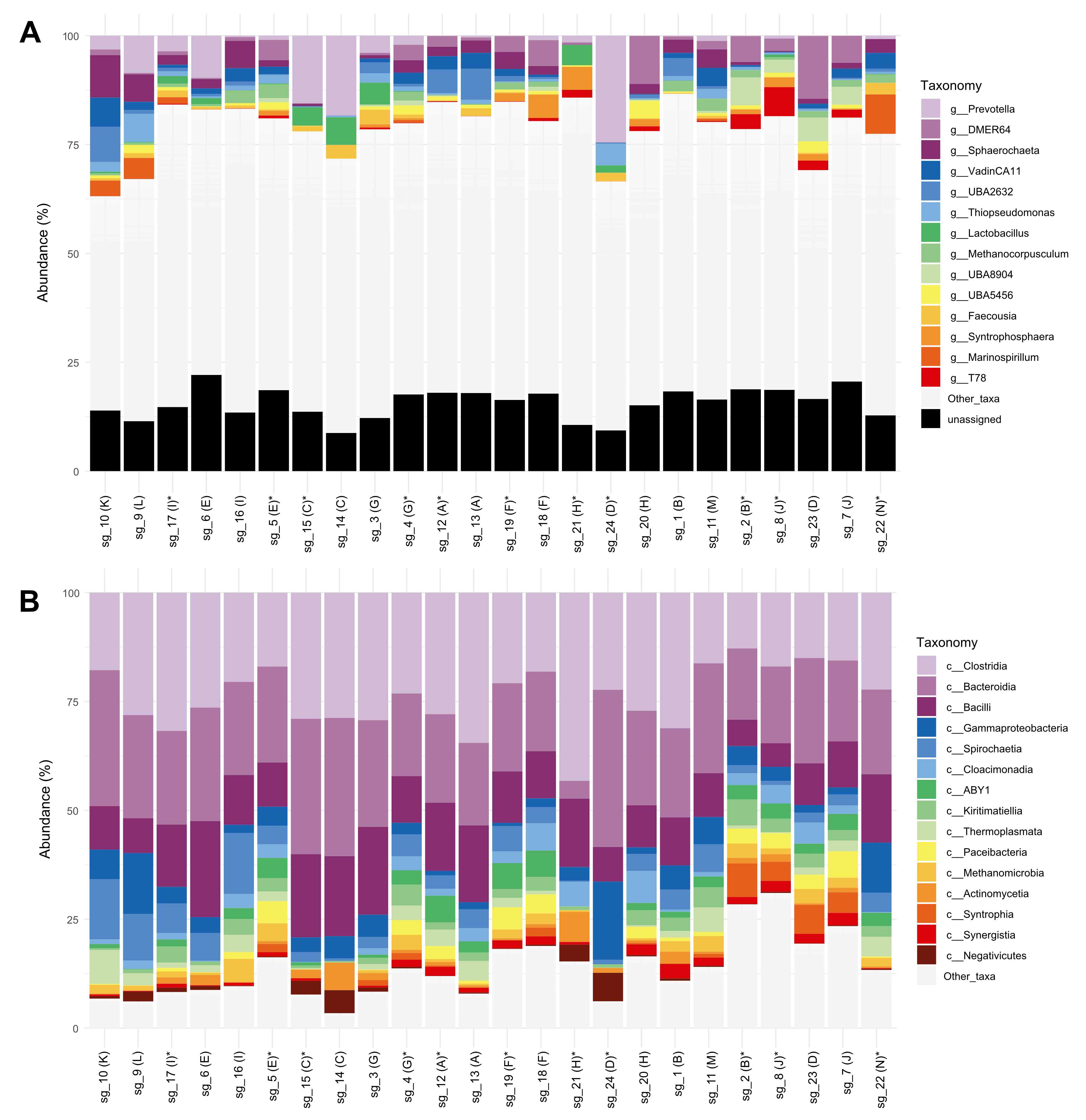
Supplementary Figure 4**. Taxonomic profiles of pig manure samples. (**A**) Dominant genera and (**B**) dominant classes, colored according to the legend. Samples collected from manure storage tanks are marked with an asterisk.

**Additional file 1**

Supplementary Table S1. Sample overview and sequencing metrics.

Supplementary Table S2. AMR gene abundances per sample normalized by estimated cell counts.

Supplementary Table S3. AMR gene abundances per sample normalized by estimated cell counts by antibiotic class.

Supplementary Table S4. AMR genes detected in metagenomic assemblies.

Supplementary Table S5. Location of AMR genes in metagenomic assemblies and distance to the closest co-located AMR or mobility-associated gene.

Supplementary Table S6. Heavy metal resistance genes detected in metagenomic assemblies within 5000 bp of AMR genes.

Supplementary Table S7. Relative abundance of bacterial and archaeal taxa determined with SingleM.

Supplementary Table S8. Antibiotic use (DCDch/animal) in 14 pig farms within six weeks and six months prior to sample collection.
